# Current approaches to the integration of sex- and gender-specific medicine in teaching: a qualitative expert survey

**DOI:** 10.3205/zma001319

**Published:** 2020-03-16

**Authors:** Katharina Clever, Cynthia Richter, Gabriele Meyer

**Affiliations:** 1 Martin Luther University Halle-Wittenberg, Medical Faculty, Institute of Health and Nursing Science, Halle (Saale), Germany

**Keywords:** curriculum, gender, gender bias, qualitative research

## Abstract

**Aim: **Although criteria and recommendations for the successful integration of sex- and gender-sensitive aspects in medical teaching have already been published, only a few medical faculties in Germany have conducted the systematic integration of sex- and gender-sensitive medicine. The aim of this expert survey, therefore, was to describe the current approaches to the integration of sex- and gender-sensitive medicine in teaching in the sense of Good Practice.

**Method: **Between April and June 2018, guided interviews were conducted with nine experts in the field of sex- and gender-sensitive medicine. Each of the experts had had experience of implementing sex- and gender-sensitive medicine at their universities. The expert interviews were then evaluated by means of quality content analysis, and frequency analyses were carried out.

**Results: **Aspects of sex- and gender-sensitive medicine were integrated both longitudinally and selectively into the compulsory curriculum or elective fields of various medical, health and nursing science courses. In the opinion of the experts, medical studies should promote the students’ gender sensitivity and in particular impart knowledge about the psychosocial and biological aspects of sex- and gender-related differences and sex- and gender-sensitive communication. For the methodological implementation of the integrated contents, didactic resources were partly adapted or developed. The players in the implementation process were confronted with various challenges, e.g. the involvement of the lecturers, the perception of sex- and gender-sensitive medicine as a women’s theme as well as ensuring the sustainable integration of sex- and gender-sensitive medicine, which is also structurally anchored in the faculty. Aspects of the curricular integration (e.g. evidence-basing, relevance in examinations) and the structural anchoring (e.g. central organization, staff availability) were mentioned i.a. as being crucial for success. A combination of top-down and bottom-up processes, e.g. by involving the faculty management but also by supporting student initiatives, was described as conducive to success.

**Conclusion:** The depicted approaches to the integration of sex- and gender-sensitive teaching contents give insight as to how sex- and gender-sensitive medicine can be integrated into the curricula. The interviews with the experts point to current themes related to sex- and gender-sensitive medicine and didactic resources. Moreover, it becomes clear which challenges are to be expected for the integration of sex- and gender-sensitive medicine in teaching and how these can be addressed. Particularly the involvement of the faculty’s lecturers but also the sustainable integration and continual quality assurance of sex- and gender-sensitive contents present challenges of a crucial nature.

## Zusammenfassung

**Zielsetzung: **Obwohl bereits Kriterien und Empfehlungen zur erfolgreichen Integration geschlechtssensibler Aspekte in die medizinische Lehre publiziert wurden, ist die geschlechtssensible Medizin bisher nur an wenigen Medizinischen Fakultäten in Deutschland systematisch integriert. Das Ziel der vorliegenden Expert*innenbefragung war es daher, im Sinne einer Good Practice aktuelle Ansätze zur Integration geschlechtssensibler Medizin in die Lehre zu beschreiben.

**Methodik: **Im Zeitraum von April bis Juni 2018 wurden leitfadengestützte Expert*inneninterviews mit insgesamt neun Akteurinnen aus dem Bereich der geschlechtssensiblen Medizin durchgeführt. Alle Expertinnen hatten an ihrer Hochschule Prozesse der Implementierung geschlechtssensibler Medizin begleitet. Die Expert*inneninterviews wurden anschließend mithilfe qualitativer Inhaltsanalyse ausgewertet und Frequenzanalysen durchgeführt.

**Ergebnisse:** Aspekte der geschlechtssensiblen Medizin wurden sowohl longitudinal als auch punktuell in die Pflichtlehre oder den Wahlbereich verschiedener medizinischer, gesundheits- und pflegewissenschaftlicher Studiengänge integriert. Nach Einschätzung der Expertinnen sollten medizinische Studiengänge die Geschlechtersensibilität der Studierenden fördern sowie insbesondere Wissen zu psychosozialen und biologischen Aspekten geschlechtsbezogener Unterschiede und geschlechtssensibler Kommunikation vermitteln. Für die methodische Umsetzung der integrierten Inhalte wurden z. T. didaktische Ressourcen adaptiert oder entwickelt. Im Implementierungsprozess wurden die Akteurinnen mit verschiedenen Herausforderungen konfrontiert, z. B. dem Einbezug der Lehrenden, der Wahrnehmung geschlechtssensibler Medizin als Frauenthema sowie der Sicherung einer nachhaltigen Integration der geschlechtssensiblen Medizin, die auch strukturell an der Fakultät verankert ist. Als erfolgskritisch wurden unter anderem Aspekte der curricularen Integration (z. B. Evidenzbasierung, Prüfungsrelevanz) und der strukturellen Verankerung (z. B. Organisationszentrale bzw. Verfügbarkeit personeller Ressourcen) benannt. Dabei wurde eine Kombination aus Top-down und Bottom-up Prozessen, z. B. durch den Einbezug der Fakultätsleitung aber auch die Unterstützung studentischer Initiativen als erfolgsfördernd beschrieben.

**Schlussfolgerung: **Die vorgestellten Ansätze zur Integration geschlechtssensibler Inhalte in die Lehre geben einen Einblick, wie geschlechtssensible Medizin in die Curricula integriert werden kann. Die Expert*inneninterviews liefern Hinweise auf aktuelle Themen der geschlechtssensiblen Medizin und didaktische Ressourcen. Darüber hinaus wird deutlich, mit welchen Herausforderungen bei der Integration geschlechtssensibler Medizin in die Lehre zu rechnen ist und wie diesen begegnet werden kann. Insbesondere der Einbezug der Lehrenden an der Fakultät, aber auch die nachhaltige Integration und kontinuierliche Qualitätssicherung geschlechtssensibler Inhalte stellen dabei zentrale Herausforderungen dar.

## 1. Introduction

Based on the biopsychosocial model, sex- and gender-sensitive medicine concerns itself with the influence of the biological sex and psychosocial/sociocultural gender with regard to the emergence, diagnosis, therapy and prevention of illnesses and has the overriding goal of ensuring the best possible healthcare for all gender [1], [2]. Thus, sex- and gender-sensitive medicine is primarily patient-oriented and should be differentiated from themes dealing with equality and promotion of women that are also part of a gender mainstreaming approach. The analysis of gender differences regarding morbidity, mortality and access to healthcare as well as the establishing of sex- and gender-sensitive treatment guidelines, prevention measures and health promotion are the key concerns of sex- and gender-sensitive medicine [3].

A necessary prerequisite for healthcare that is in keeping with the times is taking into account the variables “sex” and “gender” as important determinants of health and disease [4]. In order to avoid misuse due to different sex and gender dimensions, e.g. gender role stereotypes and to prevent gender bias in research data [5], research projects with explicit reference to sex and gender must be promoted, and in the medical faculties teaching must accordingly be further developed [6]. The ever-growing evidence-basing of sex- and gender-sensitive aspects in medicine calls for curricular integration into medical studies. Experts in the field of sex- and gender-sensitive medicine therefore recommend combining integrative and particularly explicit approaches, i.e. that sex- and gender-sensitive content is integrated as a cross-sectional issue in regular study courses as well as in courses with an explicit reference to sex and gender [7], whereby sex- and gender-sensitive themes should be included longitudinally in the courses from the first semester on. Examples of the integration of sex- and gender-sensitive medicine in Germany (Charité – Universitätsmedizin Berlin), Sweden, Canada and the USA have been described in earlier publications and the criteria of successful implementation of sex- and gender-specific aspects in medical curricula in German-speaking regions have also been summarized [8], [9], [10]. Nevertheless, the sex- and gender-sensitive content offered by medical faculties in Germany has so far been regulated very heterogeneously. In a survey conducted by the German Medical Women’s Association (Deutscher Ärztinnenbund e.V.) [11] on the state of the integration of sex- and gender-sensitive aspects, half of the faculties questioned were unable to provide information on where sex- and gender-sensitive aspects have been explicitly integrated into teaching. Just a few faculties have already integrated lectures with an explicit reference to sex and gender but these differentiate in their structural anchoring and extent of integration. Compared with international criteria [12], only one medical faculty in Germany has incorporated sex- and gender-sensitive aspects comprehensively into their teaching [11]. It must therefore be assumed that students’ sex- and gender-sensitive competences receive only little systematic support. This was confirmed by a study at two medical faculties [4] where students’ and lecturers’ knowledge about sex and gender aspects was found to be insufficient but where the significance of sex and gender aspects for the patients received wide acceptance. Therefore, the aim of the present expert survey was to record more detailed information from universities where sex- and gender-sensitive aspects have already been integrated into the studies. In line with good practice, examples of current approaches to integrating sex- and gender-sensitive aspects into teaching will be summarized.

## 2. Methods

### 2.1. Study design and sample recruiting 

In the present study, interviews were conducted with experts who had already been involved in processes implementing sex- and gender-sensitive medicine into medical, healthcare and nursing science teaching. The experts were recruited between February and May 2018. Based on literature and internet research, contacts for teaching projects in the field of sex- and gender-sensitive medicine at medical faculties were approached and experts working in the fields of sex- and gender-sensitive medicine and nursing science (e.g. [http://www.gender-curricula.com/gender-curricula-startseite/]) were contacted. In addition, experts were also recruited via calls for studies and direct contact with networks dealing with sex- and gender-sensitive medicine and gender research. Experts in the field of sex- and gender-sensitive medicine were informed about the study via e-mail. The e-mail invitation for the experts’ interviews contained information about the study objectives as well as on the themes and procedure of the interviews so that the contacted experts were also in a position to cross-check the required expertise. A total of 18 experts were invited to take part in the interviews. 

#### 2.2. Interview guideline 

The guided interviews with the experts were conducted on the telephone by the first author in the period from April to June 2018; each interview took 60-90 minutes. The interview guideline comprised five topics:

1. the structure of the integration of sex- and gender-sensitive aspects in teaching,
2. the procedure in the implementation process,
3. the integrated sex- and gender-sensitive contents,
4. the didactical realization and
5. the further development of sex- and gender-sensitive medicine in teaching.

Each topic was introduced with an open question and substantiated during the course of the interview by means of further enquiries.

#### 2.3. Data evaluation

The interviews with the experts were subsequently transcribed and anonymized. The evaluation software MAXQDA Version 10 [13] for qualitative content analyses was used to analyze the interviews using an inductive category formation for each topic [14]. If necessary, the listed categories were then summarized under main categories and the frequencies of these were then counted. 

## 3. Results

### 3.1. Expert sample

A total of nine experts from eight universities in regions where German is spoken took part in the interviews. All of the experts had either initiated processes for the implementation of sex- and gender-sensitive medicine in teaching at their university or had been significantly involved in those processes. They worked in the fields of anatomy, cardiology, plastic surgery, radiology, medical sociology, public health and nursing science or had been employed in the vice-dean’s office for studies and teaching or in the gender equality office of their university. All of the experts gave their written consent to participating in the study. 

#### 3.2. Structure of integration 

At the universities, sex- and gender-sensitive contents had been actively integrated in the following courses: Regular study course/model study course in Medicine (n=4/n=3), B.A. in Nursing and Health Promotion (n=1), M.A. in Nursing Science (n=1), Dentistry (n=3), Molecular Medicine (n=2), Public Health (n=2), M.A. in Health Professions Education (n=1), M.A. in International Health (n=1), PhD programme in Medicine/Dentistry (n=1), and in a habilitation course (n=1). 

In three universities a longitudinal integration has been achieved, for example in all the module manuals, in specific events concerning sex- and gender-sensitive medicine (compulsory modules, series of lectures) and in compulsory lectures of a “Basic Curriculum Gender Medicine”. In four other universities, sessions on sex- and gender-sensitive medicine have been selectively anchored, for example as a seminar in compulsory courses (Medical Sociology), as an elective subject, in individual modules or in a series of interdisciplinary lectures. Summarizing, four universities had a combined integration in the compulsory courses and in elective subjects or modules. Two universities had integration in the compulsory courses and one university in the elective courses. At one university an open optional workshop series had been conducted. 

The integrated aspects of sex- and gender-sensitive medicine are tested in various ways, e.g. in the semester exams (3^rd^/10^th^ semester), in a multiple choice exam at the end of the study block, as a written elective exam or a conference article (PhD). One of the universities is planning to query the sex- and gender-sensitive content in the OSCE. Moreover, one of the universities is offering the opportunity of acquiring a key qualification by completing a basic curriculum in Gender Medicine (22 compulsory lectures with integrated sex- and gender-sensitive content).

#### 3.3. Implementation process 

Various *players* were involved in the implementation process both within the universities and outside them, i.a. from the university and faculty administration, the faculty council, the academic committee, the vice-dean’s office or the study dean’s office of the Faculty of Medicine, the Equal Opportunities Officer of the Faculty of Medicine, various institutes/professorships or faculty members, as well as the Chamber of Physicians and external colleagues. 

**Figure 1:**
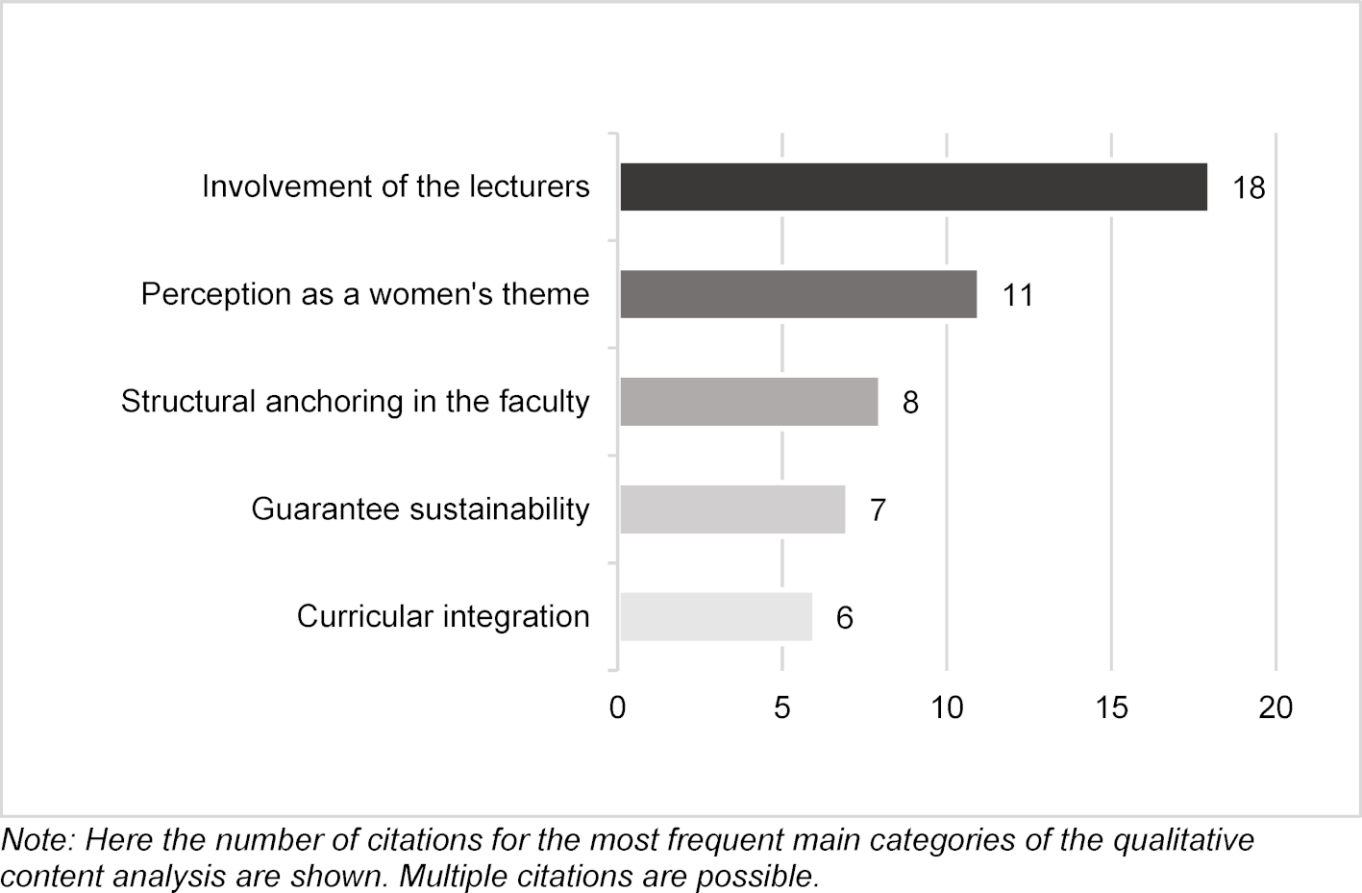
Central challenges in the implementation process

Some of the *central challenges* reported in the implementation of sex- and gender-sensitive content (see figure 1 (Fig. 1)) include the involvement of lecturers (e.g. due to a lack of interest, doubts about the relevance of the topic, or little knowledge about it); the perception of sex- and gender-sensitive medicine as a women's issue and its structural anchoring in the faculty (e.g. connection to the Equal Opportunities Office, poor networking between medicine and other specialist areas). Furthermore, the experts reported that it was difficult to guarantee the sustainability and quality of the integrated contents (e.g. due to lack of resources) and to integrate the contents in the curriculum (e.g. due to low interdisciplinarity, limited time resources in the curriculum). Apart from the central challenges shown in figure 1 (Fig. 1), the experts also mentioned uncertainties regarding the expression “sex- and gender-sensitive medicine” (n=3, e.g. confusion with equality topics), opposition from students (n=3), lack of Good Practice examples (n=2), integration in the elective sector instead of the compulsory sector (n=2), and the lack of research funding (n=1).

**Figure 2:**
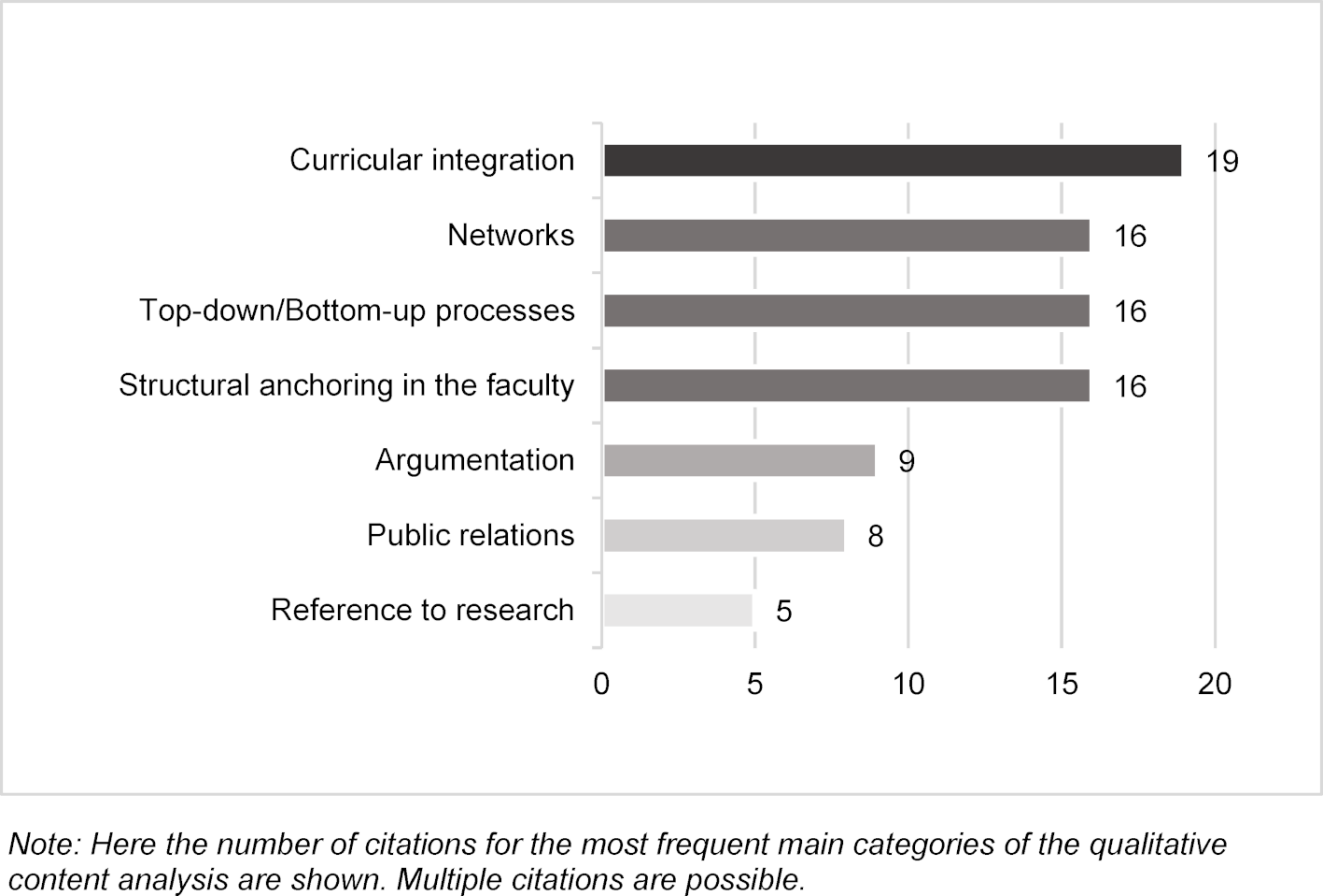
Factors in the implementation process that are conducive to success

The experts named the following aspects as *factors that were conducive to the success* of the implementation process (see figure 2 (Fig. 2)): the curricular integration (e.g. step-by-step approach, evidence-basing, learning objectives, integration in the compulsory courses, exam relevance, involvement of representatives of the medical departments), the structural anchoring (e.g. organization center, financial support), the promotion of top-down and bottom-up processes (e.g. opportunities for further and advanced medical training, involvement of the faculty administration, integration in the university development plan, support of student initiatives), the creation of networks (e.g. with the Equal Opportunities Officer), strong argumentation for the integration of sex- and gender-sensitive contents (e.g. advantages of third-party funding), broad public relations (e.g. advertising within the university, tenders, prices) and the connection to research (e.g. research promotions).

The *evaluation of the implementation process* was realized through surveys with the students (e.g. questionnaires on the specific gender curriculum two years after the initiation) or through the evaluation of teaching practices. In addition, the learning objectives formulated during the introduction of the model study programme were enumerated. Surveys with the students were also mentioned as *quality assurance measures* (e.g. talks with student representatives) as were departmental conferences with module coordinators, the sustainable safeguarding of the learning objectives, higher education didactics for lecturers, and the anchoring of the implemented contents in the directives of the system accreditation.

#### 3.4. Contents

The integrated sex- and gender-sensitive contents were often developed from the experts’ own field of expertise or from their research, were the result of benchmarking processes at other universities, or were based on surveys within the faculty or expert panels, for example with specialists or equal opportunity officers. Sex- and gender-sensitive themes were also selected according to their topicality, evidence-basing (also: Where is evidence missing at the moment?) and the possibility for reflection and discussion (e.g. gender paradox). Available catalogues of learning objectives, e.g. from the Association of Professors of Gynecology and Obstetrics (AGPO) [15] were adapted for the purpose of formulating sex- and gender-sensitive learning objectives. Table 1 (Tab. 1) presents the knowledge and skills concerning sex- and gender-sensitive medicine that the experts believe the students should attain during their studies. 

#### 3.5. Didactical realization 

Various teaching methods e.g., case studies, problem-oriented learning (POL), film material, discussions (current health-political themes), communication training, developing counseling concepts taking the variables “sex” and “gender” into account, interviews (with patients/experts), hands-on training, blended learning or research assignments were used in the lectures, seminars and working in small groups that were carried out in the study courses. Didactic resources such as the German version of the “Gender Lens Tool” [16] or the GenderMedWiki [https://gendermedwiki.uni-muenster.de/mediawiki/index.php/Willkommen_bei_GenderMed-Wiki] were applied. 

#### 3.6. Further development of sex- and gender-sensitive medicine in teaching 

With regard to the further development of sex- and gender-sensitive medicine in teaching, the experts would like the subject to be integrated systematically as an interdisciplinary topic in medical training right from the beginning of the study courses and for it to be reflected in the examinations. The individual medical departments should provide the necessary information, a specific elective would then be unnecessary. Furthermore, sex- and gender-sensitive medicine should be increasingly considered in research in order to guarantee broader empirical data. Research findings should then be transferred into teaching, and current literature and guidelines should be critically reflected.

In addition, when it comes to gender issues, medicine should become more closely networked with other disciplines, e.g. gender studies, and sex- and gender-sensitive medicine should become institutionally anchored, e.g. through a coordination office for sex- and gender-sensitive medicine. 

## 4. Discussion

Sex- and gender-sensitive medicine has already been integrated in various study courses for medicine, healthcare and nursing science, which confirms the relevance of the subject for the healthcare professions. Many of the experts have stated that sex- and gender-sensitive medicine should be understood as an interdisciplinary cross-sectional topic; however, the implementation strategies and the inclusion in the curricula have varied between the universities. Three universities achieved a longitudinal integration of sex- and gender-sensitive medicine. Here the longitudinal implementation was partly planned from the start whereas in other faculties specific teaching units on sex- and gender-sensitive medicine were selected and integrated, some of which will be replaced in the future by continuous integration in the departments or will continue to exist side-by-side. 

With regard to the integration of sex- and gender-sensitive medicine in teaching, the results of the interviews with the experts confirm the knowledge gained from other international studies. For the longitudinal integration of sex- and gender-sensitive medicine the further development of the existing curriculum is recommended, e.g. the teaching material should be checked for gender-sensitivity, gaps in the curriculum detected and integrated aspects underlined [4], [5], [6], [10], [17], [18]. In an overarching integration as a cross-sectional topic, representatives of the medical departments should select clinically relevant and evidence-based content and, if necessary, receive organizational support [10]. 

A fundamental question arises in the integration of sex- and gender-sensitive medicine into compulsory teaching or elective courses. The advantage of the elective range lies first of all in more flexible and feasible integration. On the other hand, integration would only be selective and hence reach only some of the students [19].

Incorporating the teaching staff in the integration of sex- and gender-sensitive medicine was considered by the experts as being one of the main challenges for the implementation. A lack of sex- and gender-related knowledge in the faculty, limited time resources and interest by the lecturers as well as lacking transparency about what will actually be taught have been described as obstacles in earlier publications [5], [8], [18], [19]. Authors in the field of sex- and gender-sensitive medicine therefore recommend introducing sex- and gender-related themes in faculty meetings or conferences with representatives of the medical departments, providing members of the faculty with further training in workshops or guest lectures, and making sex- and gender-sensitive teaching material available (e.g. case studies, online modules, PowerPoint slides, publication databases) [5], [6], [8], [10], [17], [18], [19], [20], [21], [22], [23]. An interesting approach to implementing the elective subject was developed at one of the participating universities: representatives from different departments were invited as speakers for the elective subject against the background of their being able to integrate the sex- and gender-sensitive contents into their own teaching in the long run.

To emphasize the focus of sex- and gender-sensitive medicine on the healthcare of all genders, this should be differentiated concerning the content, e.g. research on women’s health alone and, according to the structure, also be anchored accordingly in the faculty, e.g. not necessarily incorporated in the equal opportunities office [6], [10]. A uniform terminology and sex- and gender-sensitive language would also contribute to a more objective representation of sex- and gender-sensitive medicine [5], [10], [18]. The acquisition of third-party funding for research can also further the validation of the topic at the faculty [5]. Recommendations for the successful implementation of sex- and gender-sensitive medicine in the curricula of medical faculties are based on a fundamental commitment of the faculty as well as institutional dedication [6], [10], [17]. For this, specific structures for coordinating the integration (e.g. change agents) should be created on the one side and, on the other side, the faculty members and the students should be equally involved [5], [8], [10], [18], [22]. Moreover, the students’ motivation to learn about themes concerning sex- and gender-sensitive medicine can be encouraged by awarding additional qualifications (e.g. a key qualification in sex- and gender-specific medicine). However, the interviews with the experts showed that the integration of sex- and gender-sensitive medicine at the universities is essentially influenced by the commitment of the individual players.

Substantively, the students should, according to the experts, be sensitized first of all towards the themes of sex- and gender-sensitive medicine, particularly the psychosocial aspects of sex- and gender-related differences. For this, core competences should be suggested and registered, and should also be relevant for exams, for instance by being anchored in the German National Competence-Based Learning Objectives for Undergraduate Medical Education (NKLM) [6], [10], [18]. For the didactical realization of sex- and gender-sensitive content, the experts used primarily methods that support the students’ reflexion processes, and adapted and developed methodological resources for sex- and gender-sensitive medicine themselves (e.g. German version of the “Gender Lens Tool”, GenderMed-Wiki) [16], [https://gendermedwiki.uni-muenster.de/mediawiki/index.php/Willkommen_bei_GenderMed-Wiki].

## 5. Conclusions

The results of the interviews with the experts describe implementation approaches from various universities. The experts state that sex- and gender-sensitive medicine is a relevant cross-sectional theme in many fields and should be integrated sustainably in the curricula. In addition to a fundamental sensitization for aspects of sex- and gender-sensitive medicine (e.g. reflection on one’s own gender role, gender stereotypes), knowledge about sex- and gender-specific differences and practical skills (e.g. sex- and gender-sensitive communication) should be imparted. From the structures and success-critical factors shown in the implementation process it is possible to deduce how the successful integration of sex- and gender-sensitive medicine into teaching [7], [10], [12] can be achieved step-by-step. 

At the same time the interviews with the experts showed that the integration of sex- and gender-sensitive medicine into teaching is linked with numerous challenges. The main challenge was described as being the involvement of the lecturers, who were approached in several ways, such as by providing sex- and gender-sensitive teaching material, introducing the subject in faculty meetings or by holding advanced training courses. Problems occurred not only in the initial integration of sex- and gender-sensitive contents but also in the sustainable quality assurance of those contents, which were partly due to a shortage of human resources. The fundamental commitment of the faculty management in relation to the integration of sex- and gender-sensitive contents in the curriculum is therefore essential for success. 

## Acknowledgements

We would like to thank the experts who participated in the interviews for their support and expertise: PD Dr. med. Anja Böckers (Ulm University); Miriam Engels (Heinrich Heine University Düsseldorf), M.Sc.; Prof. Dr. phil. Margret Flieder (Evangelische Hochschule Darmstadt - University of Applied Sciences); Univ.-Prof. Dr. med. univ. Margarethe Hochleitner (Medical University of Innsbruck); Dr. rer. medic. Sabine Ludwig (Charité – Universitätsmedizin Berlin); Dr. phil. Bärbel Miemietz (Hannover Medical School); Prof. Dr. rer. nat. Dr. med. Bettina Pfleiderer (Medical Faculty of the University of Münster); Prof. Dr. med. Marianne Schrader (University of Lübeck) and Dr. phil. Simone Weyers (Heinrich Heine University Düsseldorf). Our thanks also go to the Koordinierungsstelle

Genderforschung & Chancengleichheit Sachsen-Anhalt and to the anna fischer project for helping us to recruit the experts. The authors furthermore thank Vivienne Krause for the English translation of the manuscript. 

## Competing interests

The authors declare that they have no competing interests. 

## Figures and Tables

**Table 1:**
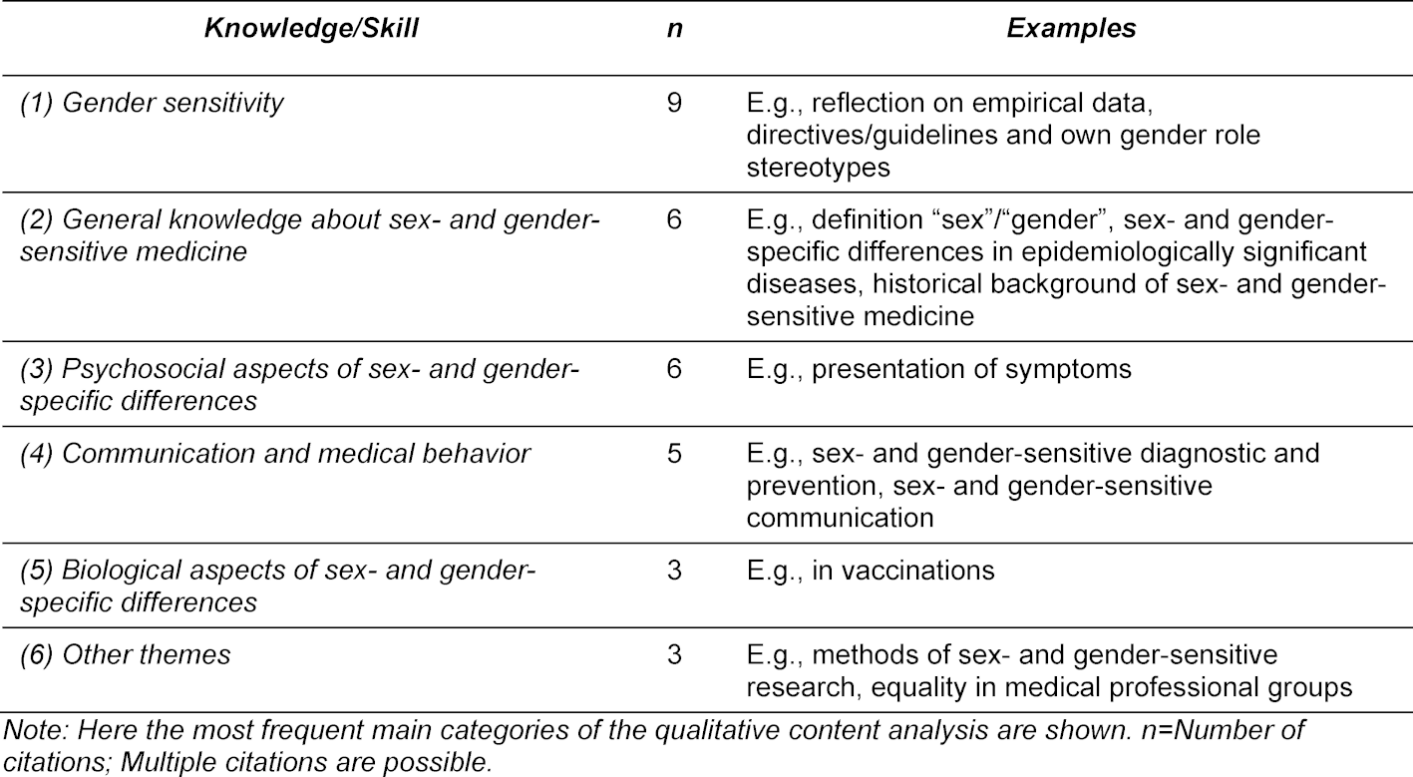
Relevant knowledge and skills of sex- and gender-sensitive medicine for students
